# Dysregulation of Transglutaminase type 2 through GATA3 defines aggressiveness and Doxorubicin sensitivity in breast cancer

**DOI:** 10.7150/ijbs.64167

**Published:** 2022-01-01

**Authors:** Gianluca Aguiari, Francesca Crudele, Cristian Taccioli, Linda Minotti, Fabio Corrà, Jeffrey W. Keillor, Silvia Grassilli, Carlo Cervellati, Stefano Volinia, Carlo M. Bergamini, Nicoletta Bianchi

**Affiliations:** 1 Department of Neuroscience and Rehabilitation, University of Ferrara, Ferrara, Italy.; 2 Department of Translational Medicine, University of Ferrara, Ferrara, Italy.; 3 Department of Animal Medicine, Production and Health (MAPS), University of Padua, Padua, Italy.; 4 Department of Chemistry and Biomolecular Sciences, University of Ottawa, Ottawa, Canada.; 5 Laboratory for Advanced Therapy Technologies (LTTA), Via Fossato di Mortara 70, 44124 Ferrara FE, Italy.

**Keywords:** Transglutaminase variants, breast cancer, GATA3, drug resistance, NC9

## Abstract

The role of transglutaminase type 2 in cell physiology is related to protein transamidation and signal transduction (affecting extracellular, intracellular and nuclear processes) aided by the expression of truncated isoforms and of two lncRNAs with regulatory functions.

In breast cancer TG2 is associated with disease progression supporting motility, epithelial-mesenchymal transition, invasion and drug resistance. The aim of his work is to clarify these issues by emphasizing the interconnections among *TGM2* variants and transcription factors associated with an aggressive phenotype, in which the truncated TGH isoform correlates with malignancy. *TGM2* transcripts are upregulated by several drugs in MCF-7, but only Doxorubicin is effective in MDA-MB-231 cells. These differences reflect the expression of GATA3, as demonstrated by silencing, suggesting a link between this transcription factor and gene dysregulation. Of note, NC9, an irreversible inhibitor of enzymatic TG2 activities, emerges to control NF-ĸB and apoptosis in breast cancer cell lines, showing potential for combination therapies with Doxorubicin.

## Introduction

Alteration of gene expression by exon skipping or use of alternative polyadenylation and splicing sites occurs frequently in cancer. In the case of breast cancer (BrCa), the affected transcripts include Breast Cancer 1 suppressor and Epidermal Growth Factor Receptor 2 (HER2) 1, along with Transglutaminase type 2 (TG2) 2, which is reported to be present at higher levels in cancer and in surrounding stroma 3 and is involved in cancer survival, spheroid production 4, resistance to chemotherapy, emergence of stem cells and epithelial-mesenchymal transition (EMT) 5-7.

The recent discovery of variants with imbalanced enzyme activities is consistent with the multiple functions of the Transglutaminase 2 gene (*TGM2*), whose expression is highly modulated by retinoic acid and other regulatory factors including TNF-α, TGF-β, NF-κB, EGF, cytokines and interleukins. Consequently, alterations of the TG2 system can affect various pathways that seem to implicate TGF-β and NF-ĸB in positive feedback loops through reciprocal mechanisms of activation 8, triggering either cell survival or death. These short remarks give reasons for our interest in the expression of the TG2 variants in BrCa cell lines of different malignancy, and their sensitivity to drugs.

The considerable size of *TGM2* and its rapidly repeated activation promote pausing of RNApol II at checkpoints along the gene during the elongation process, leading to generation of altered transcripts 9. The different isoforms encoded by *TGM2* include defective TGH2 and TGH, or alternatively spliced variants *tTGv1* and *tTGv2*, producing shorter isoforms at their C-terminal domains, affecting functions of GTP-binding/hydrolysis and nuclear translocation 10-12. Additional transcripts contain different 5'-UTR sequences, such as the variants 5 and X1, and others lack exon II or III. Finally, *TGM2* generates two long non-coding RNAs (lncRNA), TG2-lncRNA encoded from the first intron and the last exons variant (LEV) containing the terminal 3 exons and the 3'-UTR of the gene 13,14. The interest in *TGM2* transcription is supported by the evidence that isoforms are distinctly involved in EMT, tumor invasion and metastasis 6,15,16. In addition, the full-length TG2 protein supports cell survival, while the truncated TGH isoform leads to apoptosis in response to drug administration and alteration of their relative levels drives the cells towards drug-resistance 17.

In several types of cancer, drug-sensitivity can be restored using combined therapy with inhibitors of NF-ĸB, a known activator of *TGM2* expression 18, and inhibition of TG2 enhances antitumor efficacy of pharmacological molecules 19. More recently a transcriptomic study about the anticancer effects of 3-*O*-acetyl-β-Boswellic acid on MDA-MB-231 cells includes *TGM2* among the downregulated targets affecting growth, proliferation and metastasis of triple negative cancer cells 20. The role of TG2 in BrCa is further underlined by the use of short hairpin RNAs. Implantation of MDA-MB-231 cells into nude mice treated with Docetaxel produced tumors of smaller size in the group submitted to RNA interference, suggesting major antitumor effects derived by the combined therapies 21.

Some chemotherapeutic agents interact with nucleic acid sequences. This interesting aspect concerns the mechanism of action of Doxorubicin (Doxo), which is able to bind selectively DNA interfering with chromatin packaging and leading to modulation of transcription 22,23, with an increased production of alternatively truncated polyadenylated variants, as described for Topoisomerase II and other genes that regulate the cell cycle 24. Note that Doxo induces persistent activation of TG2 in association with cell survival and transamidation activity of the full-length enzyme, which is crucial in Doxo-resistant phenotypes 25.

Our study on BrCa cell lines serves the purpose to provide a model to unravel the modulation of altered *TGM2* transcriptional variants following pharmacological exposure and to study the effects of their changes in association with transcription factor interactions, among them GATA3 looks to play a pivotal role. We think that the different expression of altered *TGM2* transcripts should be investigated as predictive marks to drive combined treatments towards improving efficacy and overcoming chemo-resistance.

## Materials and Methods

### BrCa Cell culture and drug treatments

Experiments were carried out on the human hormone responsive MCF-7 and T47D epithelial cell lines (ER+/PR+/HER2-), and on the triple-negative MDA-MB-231 from adenocarcinoma cells that differently from the others display a mesenchymal-like phenotype. All cell lines derive from strains obtained from American Type Culture Collection (Rockville, MD, USA). MCF-7 cells were cultured in Dulbecco's modified Eagle's medium (DMEM, GE-Healthcare, Milano, Italy) supplemented with 10% foetal bovine serum (FBS) and 2 mM L-glutamine (Sigma-Aldrich, St. Louis, MO, USA), T47D in RPMI supplemented as described above, and MDA-MB-231 in L15 medium, 2 mM Glutamine and 15% FBS. Antibiotic solutions were added to the cultures at concentration of 50 U/ml penicillin and 50 μg/ml streptomycin (Sigma-Aldrich), incubated at 37°C under an atmosphere of 5% CO_2_ in humidified air. MDA-MB-231 were plated at a density of 2x10^5^ and MCF-7 and T47D at 3x10^5^ cells/cm^2^. Cells were treated with the anticancer drugs (purchased from Chemietek, Indianapolis, IN, USA) dissolved in 0.1% DMSO and employed at the final concentrations of 1 µM AZD5363, 1 µM BYL719, 1 µM Gefitinib, 2 μM Doxo, 0.1 µM Docetaxel and 1 µM XL765. Notably, AZD5363 is an inhibitor of PKB/AKT isoforms, BYL719 inhibits specifically the PI3K α-isoform, Gefitinib targets Epidermal Growth Factor Receptor tyrosine kinases, Doxo binds DNA inhibiting the activity of Topoisomerase II, Docetaxel binds tubulin and induces cell-cycle arrest at the G2/M phase in addition to inhibit Vascular Endothelial Growth Factor, while XL765 is a reversible ATP-competitive inhibitor of pan-Class I PI3Kα, β, γ, δ and mTORC1/mTORC2.

In synergic treatment, the TG2 inhibitor NC9 was added at 30 μM, after 24 h of administration of 0.2, 0.5 or 2 μM Doxo, in double combination for additional 16 h, until the cells were assayed for apoptosis using Annexin V, for cytotoxicity using Cell&Viability kit and Cell Cycle Kit by the MUSE cell analyzer (Luminex Corporation, Austin, TX, USA). At the end of treatment, TG2 was analysed by Western blot using a specific antibody Ig036 (Zedira, Darmstadt, Germany) recognizing both TG2 and TGH, as reported 14.

### Extraction of RNA and quantification of gene expression by reverse transcription and quantitative PCR reactions

About 3×10^6^ cells, collected by centrifugation for 10 min at 1200 rpm at 4 °C, were washed three times in phosphate buffered saline (PBS, ThermoFisher Scientific, Invitrogen, Monza, Italy) and total RNA was extracted by TRI Reagent^®^, following instructions provided in the manufacturer's protocol (Sigma-Aldrich). Reverse transcription was performed using 1 µg of total RNA and TaqMan® Reverse Transcription Reagents kit (ThermoFisher Scientific) with primers and conditions of quantitative polymerase chain reaction (qPCR) reported in Franzese *et al.* 14 using hypoxanthine phosphoribosyl transferase 1 (HPRT1) as reference gene 26. Fold change was determined by comparing the threshold cycle relative value (CT) of target gene with that of the amplified HPRT1 reference to obtain ΔCT value. To quantify the increase we employed the differences between ΔCT of the treated and untreated samples, the negative exponent in the formula 2^-ΔCT^ to express target modulation, and the 2^-ΔΔCT^ to quantify fold change in a comparative manner.

### Arrested-PCR at putative regulatory target sequences by *in vitro* incubation with Doxo

Target sequences of both *TGM2* promoter and intron 10 are reported in Supplementary Table 1 along with the size of PCR products and primers of each template used. Exonic HPRT1 fragment was employed as a negative control. PCR fragments were incubated 10 min with increasing concentrations of Doxo (1, 5, 10, 25 and 50 μM), then amplified by PCR with SYBR® Green Master Mix (ThermoFisher Scientific) and products were checked by electrophoresis in 1% agarose gel with 40 mM Tris-acetate, 1 mM EDTA (TAE) buffer, pH 8.3.

### GATA3 silencing and induction by Doxo

The silencing of MCF-7cells already expressing GATA3 was performed without stimulation by Doxo, which instead was employed at 2 μM for 18 h to induce GATA3 in the MDA-MB-231 cells. Before silencing, medium was removed from each well and 800 μl of antibiotic and FBS free-medium were added along with 200 μl of siRNA transfection solution. For silencing, BrCa cells seeded according to the protocol of Dharma FECT (Carlo Erba Reagents, Milan, Italy) were transfected with siRNA against GATA3 transcript (Individual ON-TARGETplus GATA3 siRNA, cat. FE5J003781060002) and negative control siRNA (siRNA neg, cat. FE5D0013200120). The lyophilized siRNAs were resuspended at 5 μM concentration and diluted to 100 nM final concentration 27 for experiments in 12-wells plates (4 cm^2^/well). After 8 h of incubation FBS was added and 48 h later RNA was extracted and GATA3 expression was analyzed by reverse transcription and quantitative polymerase chain reaction (RT-qPCR) using 150 nM primers (FGATA3Ex, 5'-CAGCACAGAAGGCAGGGAG-3' and RGATA3Ex, 5'-TCTGACAGTTCGCACAGGAC-3') and PowerUp™ SYBR^®^ Green Master Mix (ThermoFisher Scientific, Invitrogen, Monza, Italy) in 20 µl of reaction mixture.

### Datasets from GEO for *TGM2* gene expression analysis

For our study of *TGM2* gene expression, we extracted data from public GEO datasets and analyzed them using HumanExon1_0ST Affymetrix. This array panel is formed by specific probes distributed along the whole genome, allowing rigorous quantification of the expression of all altered transcripts.

The first dataset (access number GSE58598, available on-line at https://www.ncbi.nlm.nih.gov/geo/query/acc.cgi?acc=GSE58598, 18 August 2021) employed in this study includes FFPE tissue derived from biopsies. The epithelial and stromal cells were obtained using Laser Capture Microdissection from BrCa of patients. The second dataset (access number GSE16732, available on-line at https://www.ncbi.nlm.nih.gov/geo/query/acc.cgi?acc=GSE16732) includes samples derived from 40 types of BrCa cell lines. These included 16 ER+ cell lines (ZR75-30, ZR75-1, BT474, MDA-MB-361, UACC812, SUM44, MCF-7, T47-D, CAMA-1, BT483, MDA-MB-415, MDA-MB-330, MPE600, SUM52, MDA-MB-134VI, MDA-MB-175VIII); 8 ER-/HER2+ cell lines (EVSA-T, OCUB-F, SK-BR-3, SK-BR-5, SUM190, SUM225, MDA-MB-453, UACC893) and 16 ER-/PR-/HER2- triple negative cell lines (MDA-MB-468, BT20, DU4475, HCC1937, MDA-MB-436, SUM185, SUM229, Hs578T, OCUB-M, MDA-MB-157, MDA-MB-231, SUM1315, SUM149, SUM159, BT549, SK-BR-7).

The *TGM2* gene expression analysis of these datasets was performed by normalizing signal intensity (NI) for each specific probe of the variants and comparing them among the samples, as described by Minotti *et al*. 28.

## Results

### Overexpression of TGH transcript as mark of aggressive phenotype

**Figure 1:**
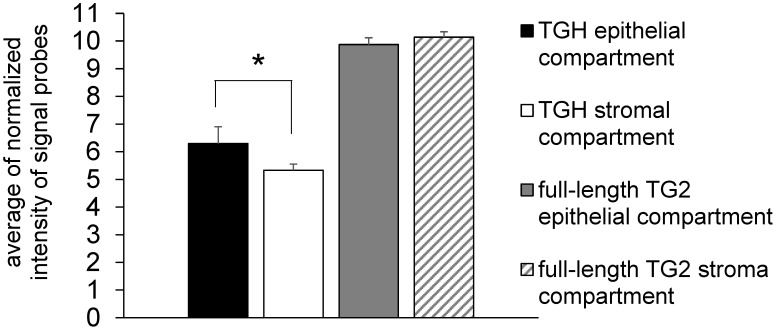
** Expression of full-length TG2 and TGH in epithelial and stromal tissues.** Analysis with HumanExon1_0ST Affymetrix array of the transcripts from dataset GSE58598 is quantified on the base of Normalized Intensity (NI), in FFPE samples derived from biopsies of patients affected by BrCa (epithelial n=6, stromal n=4). *p*<0.02 is marked by an asterisk for TGH, calculated with GraphPad Prism 6, using unpaired *t* test two-tailed comparing the epithelial *vs.* stromal compartment.

Since many chemotherapeutic agents trigger dysregulated transcription of genes involved in cell cycle 24, we have focussed our studies on the levels of *TGM2* transcripts in BrCa taking into account the cell distribution of the TG2 transcripts. In preliminary experiments we analyzed gene expression in samples derived from epithelial (n= 6) and stromal (n= 4) components of formalin-fixed paraffin-embedded (FFPE) tissue sections by means of the accessible dataset of GEO (GSE58598, as detailed above) using the Affymetrix GeneChip® Human Exon 1.0 ST array. The expression of full-length TG2 transcript did not display differences between epithelial and stromal compartments, while TGH mRNA significantly diverged with higher expression in the epithelial compartment (*p*<0.02) (Figure 1).

**Figure 2:**
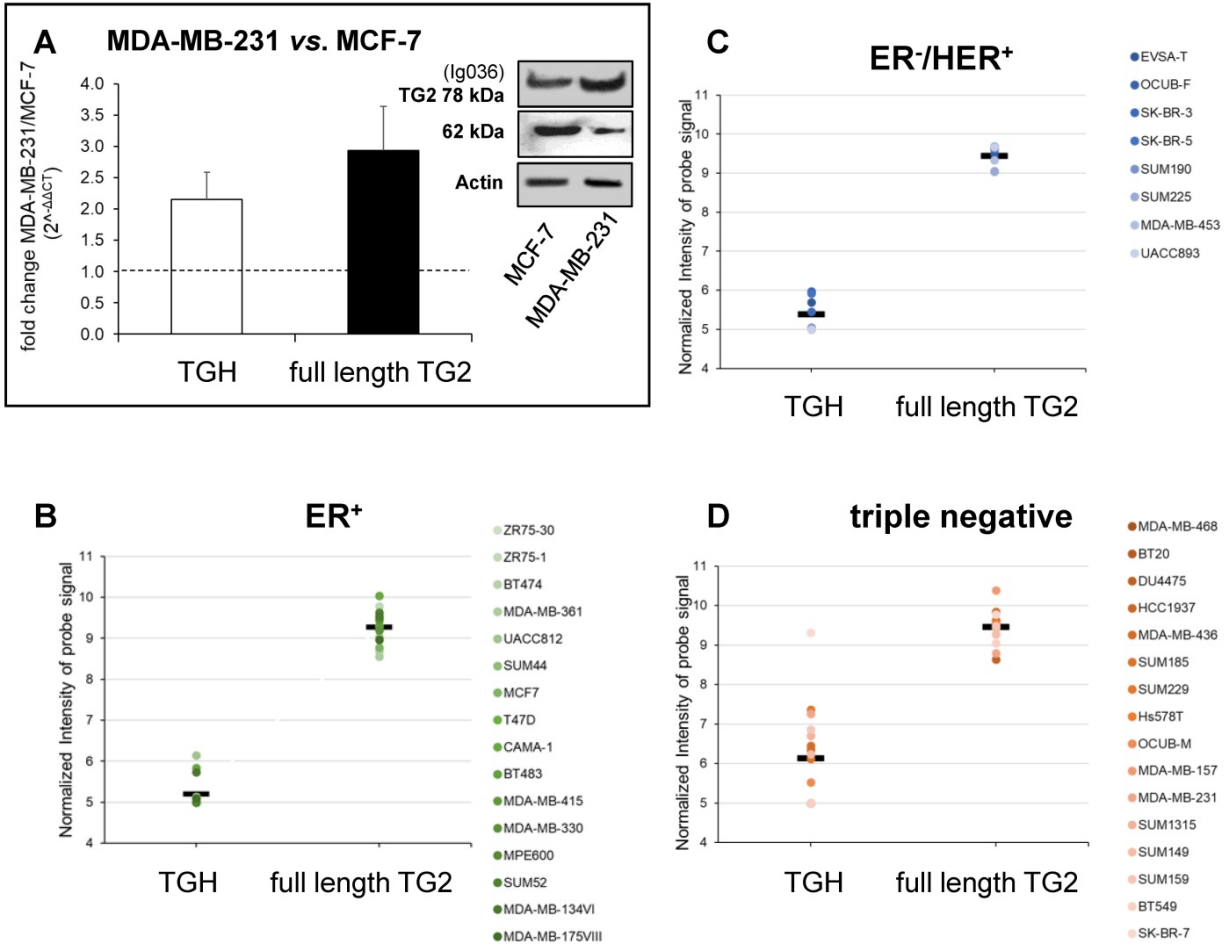
** Analysis of *TGM2* expression in BrCa cells with different phenotypes.** In panel **A** are presented the values of proteins analysed by Western blot, the canonical TG2 isoform of 78 kDa evidenced using mouse monoclonal antibody against β barrel 1 domain (Ig036, Zedira, Germany) and actin as reference; in addition, the full-length TG2 and TGH transcripts were quantified by RT-qPCR comparing MDA-MB-231 to MCF-7 cells as reference sample. Fold increase was evaluated using 2^-∆∆CT^ with HPRT1 as housekeeping gene and *p*<0.05 calculated with GraphPad Prism 6, using unpaired *t* test two-tailed. Panels **B**, **C**, **D** report gene expression analysis from the GSE29682 dataset of GEO database clustered in ER+, ER-/HER2+ and triple negative phenotypes. Full-length TG2 and TGH transcripts are quantified on the base of Normalized Intensity (NI) of probe signal recognizing the specific variant as obtained with the HumanExon1_0ST Affymetrix array. The mean values are represented by a bar and each cell line is represented by a point. P values for the full-length TG2 and TGH were calculated with GraphPad Prism 6 comparing the 3 groups to each other. For TGH the comparison ER+ vs. triple negative has *p*<0.006 using unpaired *t* test two-tailed, while ER-/HER2+ *vs.* triple negative has *p*<0.035 using unpaired *t* test and Welch's correction.

We have further extended the analysis of full-length TG2 and TGH to BrCa taking into account the cell lines MCF-7 (ER+/PR+/HER2-) that can be induced to EMT by drug treatments assuming mesenchymal features, and MDA-MB-231, representative of triple negative signature (ER-/PR-/HER2-), invasive and highly aggressive. Data from RT-qPCR analysis showed higher levels of both transcripts in MDA-MB-231 cells compared to MCF-7 cells at 48 h of culture (Figure 2A), in agreement with the greater content of TG2 protein evidenced by Western blot in the triple negative cells (panels of Figure 2A).

To verify any association between upregulation of *TGM2* and aggressive phenotype, we extended the investigation to consider gene expression profiles extracted from the GSE16732 dataset of 40 cell lines 29,30 (including the 16 ER+, 8 ER-/HER2+ and 16 ER-/PR-/HER2- cell lines as defined in Material and Methods section) characterized by a different expression of diagnostic markers routinely employed in histological typing of the tumour. Differences in the levels of the full-length TG2 transcript were moderate within the different samples, while TGH, less expressed than full-length TG2, significantly accumulated in triple negative cells. This was verified by the normalized values of the specific probe recognizing TGH that increased progressively from 5.199 ± 0.09 in ER+ cell lines (above average in 3/16, Figure 2B), to 5.387 ± 0.133 in ER-/HER2+ (increased in 5/8, Figure 2C), and finally in triple negative phenotype with the highest value of 6.137 ± 0.302 (increased in 9/16, Figure 2D). These differences of TGH expression were statistically significant, with *p*<0.006 when comparing the ER+ *vs*. triple negative phenotype (determined by GraphPad Prism 6 using unpaired *t* test assuming two-tailed Gaussian distribution and the same SD in the populations) and *p*<0.035 when comparing ER-/HER2+ *vs*. triple negative phenotype (applying Welch's correction). We underline that among the sixteen triple negative cell lines, six derived from primary tumours (BT20, HCC1937, Hs578T, SUM149, SUM159, BT549) had no significantly different expression of TGH transcript when compared to those isolated from pleural effusion having metastatic origin.

### Induction of *TGM2* transcription by chemotherapeutic drugs in MCF-7 and MDA-MB-231 cells. Specific effects of Doxo on TGM2 promoter and sequences in intron 10

**Figure 3:**
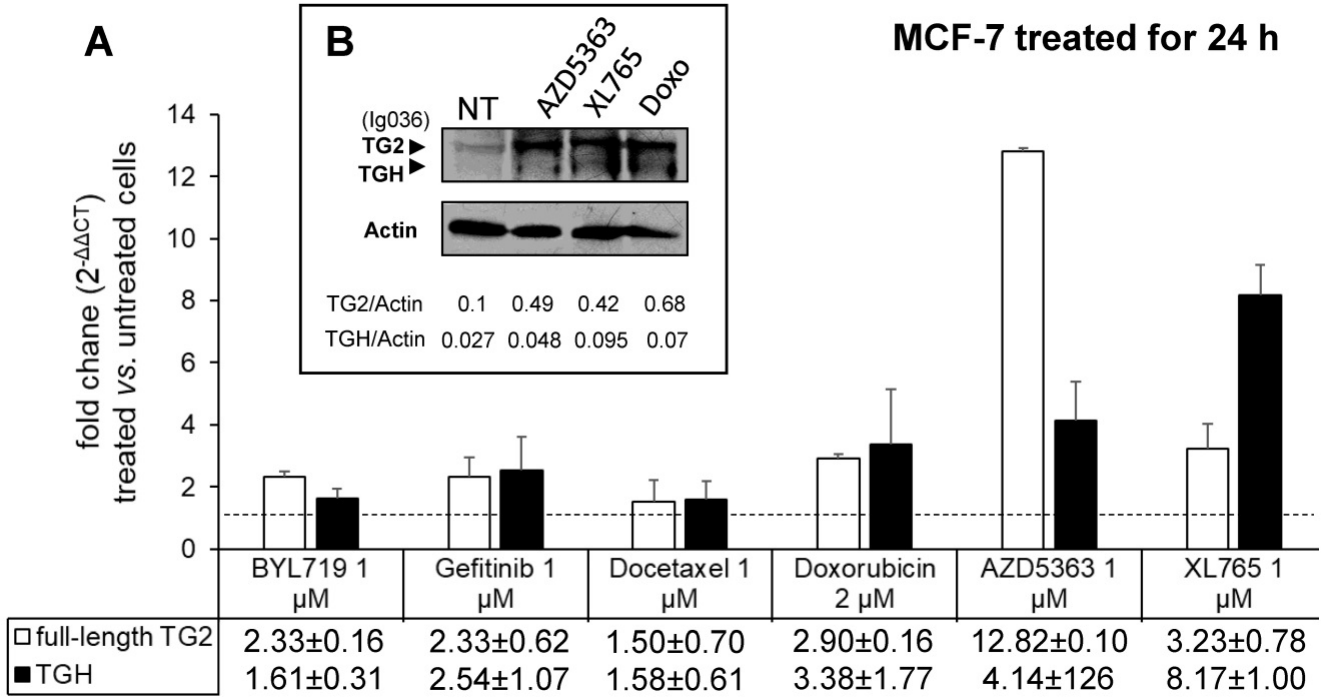
** Analysis of *TGM2* expression in MCF-7 cells treated with anticancer drugs.** In **A**, after treatment with the listed compounds for 24 h, fold increases of full-length TG2 and TGH mRNAs were quantified by RT-qPCR using 2^-∆∆CT^ with HPRT1 as reference gene and compared to cells treated with solvent (0.1% DMSO) as control. Average value and SD were calculated from n= 3 independent experiments (ANOVA, *p*<0.05). In **B**, protein levels of full-length TG2 and TGH are reported with the ratio values, obtained by exposure to the most effective drugs and analyzed by Western Blot using mouse monoclonal antibody against β barrel 1 domain (Ig036, Zedira, Germany) and actin as reference.

**Figure 4:**
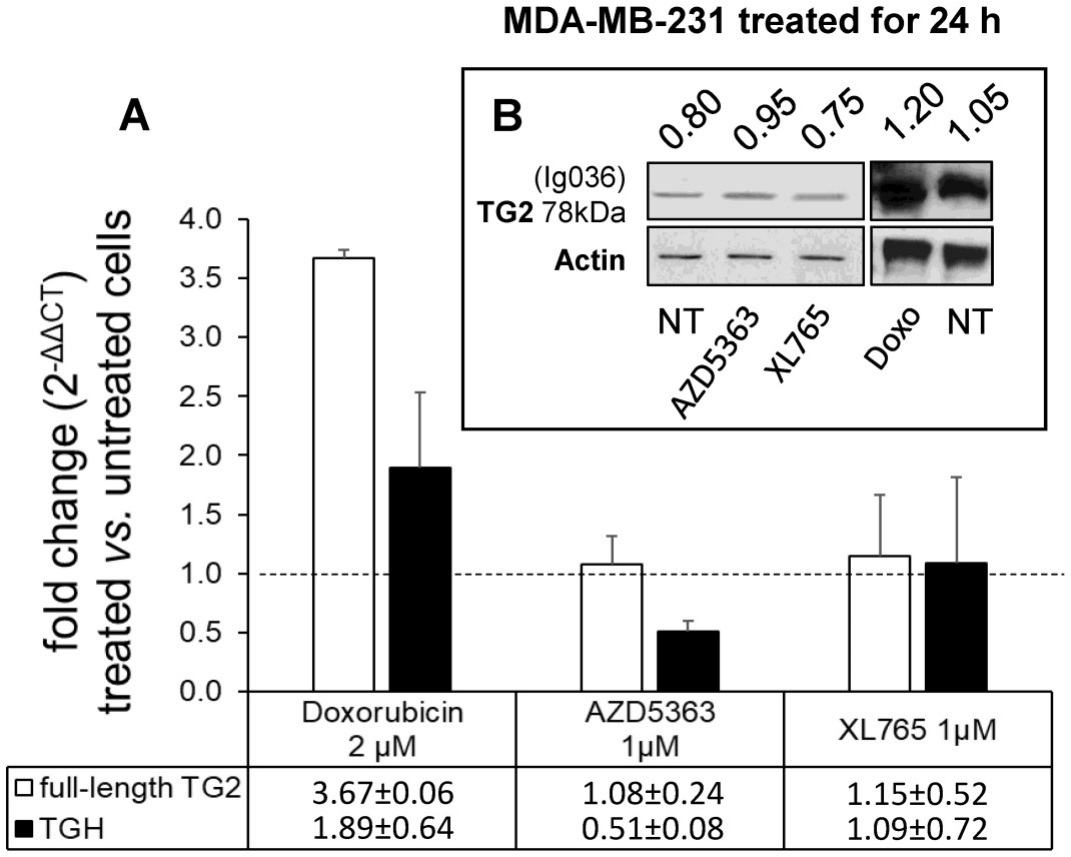
** Analysis of *TGM2* expression in MDA-MB-231 cells treated with anticancer drugs.** In **A**, after treatment with the listed compounds for 24 h, fold increase of full-length TG2 and TGH transcripts were quantified by RT-qPCR using 2^-∆∆CT^ with HPRT1 as reference gene and compared to cells treated with solvent (0.1% DMSO) as control. Average value and SD were calculated from n= 3 independent experiments (ANOVA, *p*<0.05). In **B**, protein amount of full-length TG2 is reported with the ratio value calculated with respect to actin as reference, analyzed by Western Blot using mouse monoclonal antibody against β barrel 1 domain (Ig036, Zedira, Germany).

To evaluate the drug sensitivity of the dysregulated expression of TG2 variants in ER+ and in triple negative BrCa cell lines, we performed experiments on MCF-7 and MDA-MB-231 cells. In the first instance, we exposed MCF-7 cells for 24 h to anticancer drugs with different mechanisms of action (AZD5363, BYL719, Gefitinib, Doxo, Docetaxel, and XL765 each at its IC_50_ concentration) quantifying the levels of full-length TG2 and TGH by RT-qPCR and we detected an increase of transcripts following treatment with all drugs (Figure 3). In parallel, we noted higher levels of TG2 protein after these treatments in this type of cells that easily evolve towards EMT. The most significant effects were observed for AZD5363, XL765 and Doxo, which also increased a band corresponding to TGH by Western blot. We performed an analogous study on the triple negative MDA-MB-231 cells (which have significantly higher basal starting levels of TG2) using the more active compounds AZD5363, XL765 and Doxo). The results demonstrated that only Doxo was able to induce the expression of both *TGM2* transcripts and that the amount of TG2 protein varied slightly after 24 h of exposure (Figure 4).

We have further focused our study on the effects of Doxo with respect to the transcriptional expression of TG2 isoforms because its mechanism of action involves recognition of specific DNA sequences and interference at transcription binding sites that could lead to occurrence of altered transcripts 22,24, as well as the suppression of transcriptional/duplication processes through inhibition of Topoisomerase II.

Studies on epigenetic modifications at *TGM2* promoter in several types of cancer have demonstrated that the methylation status 31,32 and Myc-mediated histone deacetylation modify the expression of the gene 33. Among the reliable Doxo target sequences, we investigated four putative regulatory regions of *TGM2* that are epigenetically silenced in BrCa 34 and contain consensus sequences for transcription factors associated with Doxo-resistance through SNPs at the promoter 35. Among them, the PCR1 template (332 bp) contains an ELF-1 site (chr20:36,797,343-36,797,618 GRCh37/hg19) directly involved, while the PCR2 (578 bp) includes Sp1 (chr20:36,796,357-36,796,792 GRCh37/hg19), which displays only a regulatory role, and an additional ELF-1 site (chr20:36,796,361-36,796,696 GRCh37/hg19). The region denoted fragment PCR3 (549 bp), across TG2-lncRNA in the intron 1, includes the transcription start site, a third ELF-1, RNApol II and GATA3 sites (chr20:36,793,632-36,794,015 GRCh37/hg19) in addition to a CpG island (chr20:36,793,550-36,793,867 GRCh37/hg19) 14. The last PCR4 fragment (141 bp) is inside the intron 10, located after the splicing site and contained consensus sequence target of GATA3 (chr20:36,763,169-36,763,309 GRCh37/hg19), also recognized by a RNApol II site in other type of cancer 14.

**Figure 5:**
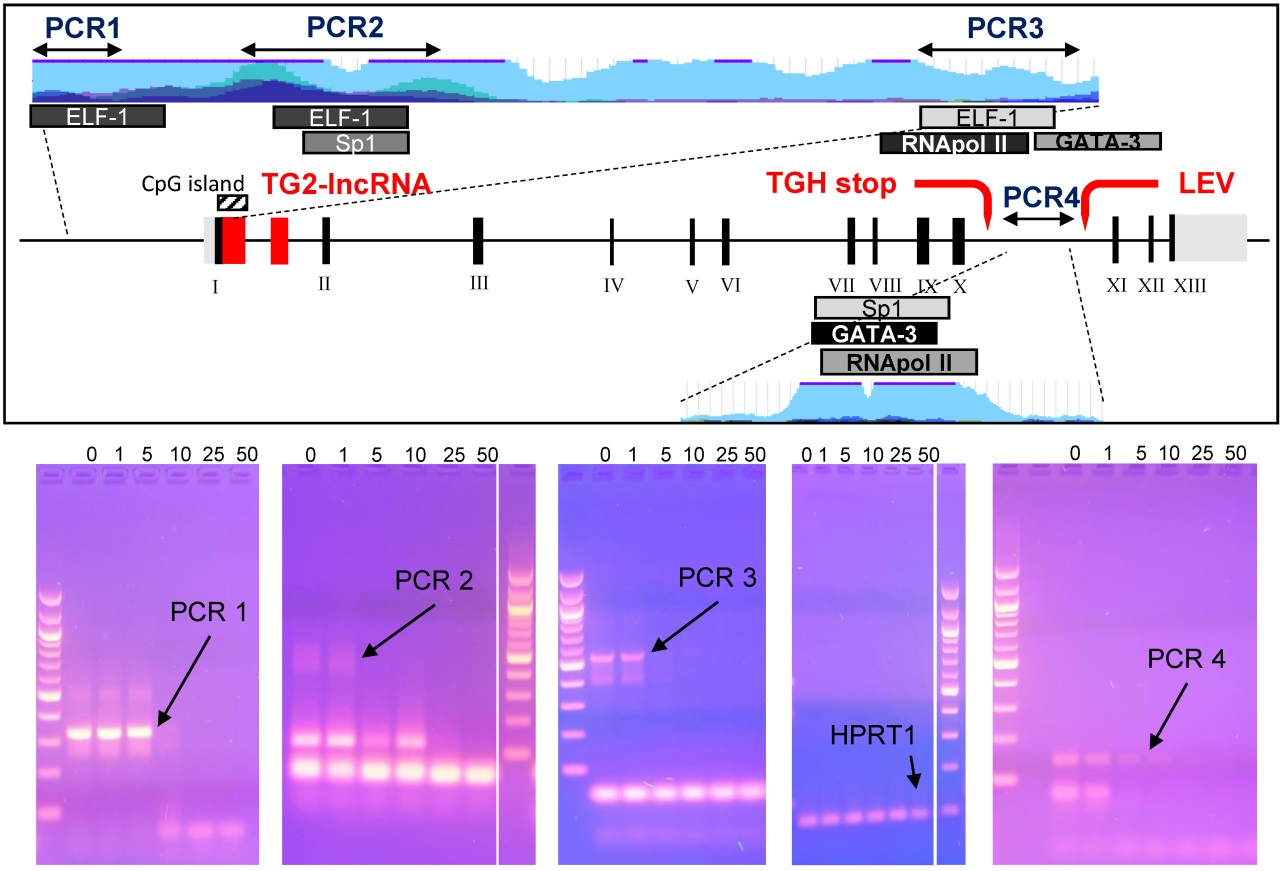
** In the box the *TGM2* gene map, with the numbered exons in Black, while in Red the TG2-lncRNA within the intron 1, the stop codon generating the truncated variant TGH and the start of transcription of another lncRNA named LEV.** The portions of the gene amplified by arrested-PCR were enlarged with positions of the selected transcription factors and CpG island, as well as H3K27Ac marks (light blue). In Blue, regions corresponding to the arrested-PCR1, -PCR2, -PCR3 of *TGM2* regulatory regions, and -PCR4 of intron 10. In the bottom, the PCR amplifications without preincubation with drug (0), or carried out following 10 min incubation with 1, 5, 10, 25 and 50 μM of Doxo. Amplification conditions are specified in Material and Methods and described in Supplementary Table 1. The PCR amplified from an exonic region of HPRT1 was used as negative control to test Doxo-binding. Electrophoretic migration was performed in 2% agarose gel in TAE-Buffer. Molecular size marker used is 100 bp DNA ladder (New England Biolabs, MA, USA).

We tested *in vitro* interference by Doxo incubation on these PCR products for 10 min before amplification 36, using progressively increasing concentrations of 1, 5, 10, 25 and 50 μM. We have chosen exonic HPRT1 fragment as a negative control to determine the specificity of the DNA-binding drug. PCR products, primers and sizes are summarised in Supplementary Table 1. Doxo inhibited the amplification of the DNA templates PCR1, PCR2, PCR3 and PCR4, but not of the HPRT1 fragment (Figure 5, bottom panel). Appreciable effects were observed on PCR1 and PCR4 with reduction of intensity at 5 μM, and on PCR2 and PCR3 at as low as 1 μM. These results suggest that Doxo affects the *in vitro* elongation process. Highly interesting are the effects on PCR3 containing CpG island, subject to methylation by DNA methyltransferase I known to be inhibited by Doxo, the activity of which was recently reported to alter profile of cell survival genes 37.

Our selected regulatory regions were analysed using ChIP-sequencing datasets from ENCODE carried out on MCF-7 cells to verify the presence of binding sites for the nuclear factors indicated in Figure 5. In the relevant data summarised in Supplementary Figure 1, we identified high signal peaks of enriched-reads mapping to sequences recognized by RNApol II, adjacent to a less pronounced one for GATA3 present in the PCR3 fragment. The same genomic region is recognized by di- and tri-methylated H3K4 in correspondence with the CpG island encoding TG2-lncRNA, inside the intron 1 close to the transcription starting point, where H3K4me2 is associated to transcriptional activation of genes promoting DNA repair deriving from genotoxic stress 38,39.

An additional GATA3 binding site, shown in Supplementary Figure 2, displayed remarkably high enrichment at the GATA3 binding site in the intron 10 (PCR4 sequence), in a regulatory region downstream the alternative stop codon generating TGH and upstream of the start of the second LEV lncRNA, already described in other types of cancers by Franzese *et al*. 14. This latter GATA3 consensus element is evolutionarily conserved and corresponds with enrichment peaks evidencing histone marks like H3K4me1 or H3K27ac, the last requiring additional factors to coordinate enhancer functions 40. The signal intensity of this second peak at the intron 10 (PCR4) is in agreement with its higher recognition by GATA3 than that located in the intron 1 near the start of transcription (PCR3). This later position will be also targeted by GATA3 in the presence of major levels of expression of the factor.

### GATA3 interferes with accumulation of TG2 isoforms

**Figure 6:**
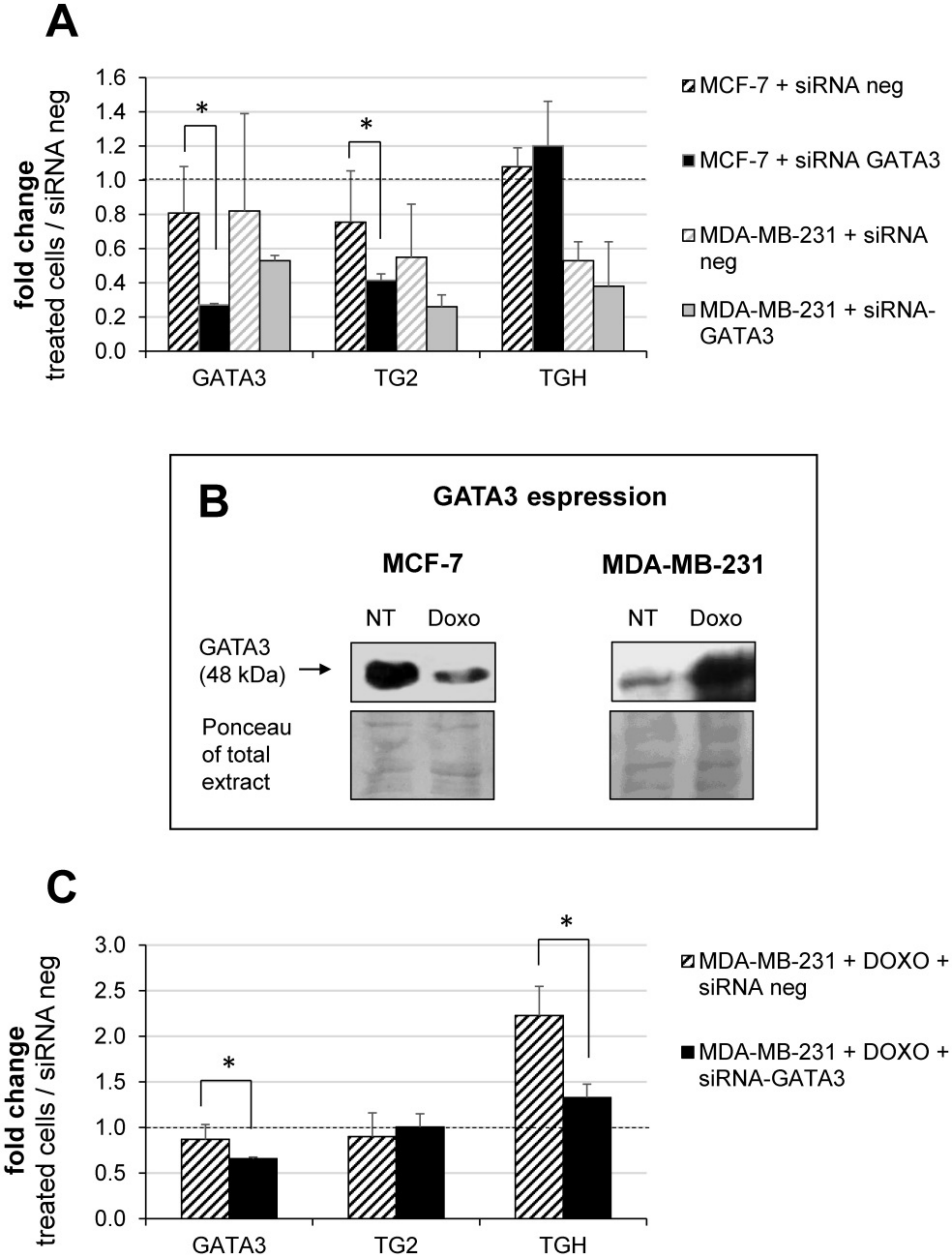
** Modulation of GATA3 expression by siRNA molecules.** In **A**, silencing of GATA3 using 100 nM of specific and negative control siRNA carried out in MCF-7 and MDA-MB-231 cells. RT-qPCR analysis of GATA3, full-length TG2 and TGH mRNAs evaluated referring to another sample treated with siRNA neg, taken as control. Formula 2^-∆∆CT^ and HPRT1 as reference gene were used to obtain the fold change. *p*<0.01 indicated with asterisk was calculated using GraphPad Prism 6, unpaired *t* test two-tailed (n= 3 independent experiments). In **B**, Western blot analysis of extract from MCF-7 and MDA-MB-231 cells untreated or treated with 2 μM Doxo. GATA3 was detected by hybridization with rabbit monoclonal antibody ([EPR16651] ChIP grade ab199428 purchased from Abcam). Ponceau S stain is shown to compare the protein amounts in the extracts. In **C**, silencing of GATA3 using 100 nM of specific and siRNA neg carried out in MDA-MB-231 cells pre-treated with 2 μM Doxo (as reported in Material and Methods). Quantification by RT-qPCR of GATA3, full-length TG2 and TGH mRNAs was evaluated referring to another set of samples treated with siRNA neg. HPRT1 was used as reference gene to apply formula 2^-∆∆CT^. Fold change was reported and *p*<0.001 indicated with asterisk was calculated using GraphPad Prism 6, unpaired two-tailed *t* test (n= 4 independent experiments).

Building on our previous report on the *TGM2* in other tumours and on the effects of retinoic acid which involves GATA3 14, we decided to investigate the modulation of the *TGM2* response because GATA3 interacts both with the promoter and additional binding/pausing sites within the intron 10. In BrCa, expression of GATA3 correlates with tumour differentiation, as it is more expressed in MCF-7 41 and controls invasiveness of MDA-MB-231 cells 42-44. In this perspective, we have analyzed whether the levels of TG2 and TGH change after silencing of GATA3 using siRNA compared to treatment with siRNA negative control. Data quantified with respect to a set of other siRNA neg samples are reported in Figure 6A, from which it emerges that GATA3 silencing was significantly effective only in the MCF-7 cells. This correlated with a decline in the amount of full-length TG2 transcript, but not in TGH, according with lower affinity of the consensus sequence in intron 1 than that inside the intron 10, which displays high signal in the enrichment peak (comparison between Supplementary Figure 1 and 2). This evidence confirms the persistence of GATA3 at that position and the zone of the stalling of RNApol II, promoting the use of alternative stop site upstream coding TGH. In contrast, MDA-MB-231 cells contain very low basal-levels of GATA3, so silencing cannot be performed. For these reasons, we have verified GATA3 expression in the presence or in the absence of Doxo (Figure 6B, in the box). Our findings indicated that the amount of GATA3 protein decreased after treatment in MCF-7, but was markedly increased after exposure to the drug in MDA-MB-231 cells (Figure 6B). Thus, silencing GATA3 in MDA-MB-231 under stimulation with Doxo significantly reduced TGH expression without affecting the level of full-length TG2 (Figure 6C), whose promoter was stably demethylated and insensitive to the pharmacological action. Also, CpG island at intron 1 results completely demethylated 35 and the regulating elements in that position will be more accessible to other transcriptional control factors.

### Effects of Doxo-mediated TG2 induction were limited by NC9 inhibitor

**Figure 7:**
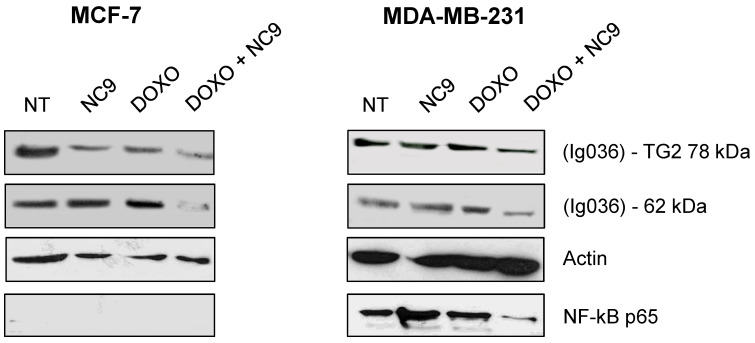
** Effects of treatment with NC9 and Doxo on NF-ĸB expression.** Exposure to Doxo was carried out during 40 h. At 24 h the NC9 was added at 30 μM concentration, and treatment was continued for additional 16 h. Western blot analyses were carried out in MCF-7 and MDA-BM-231 cells (right left panel respectively). The electrophoretic run was optimized to discriminate possible full-length TG2 of 78 kDa with a specific Ab directed to terminal carboxy portion of the protein (Ig037, Zedira, Germany) and TGH as additional band of 62 kDa evidenced by using of mouse monoclonal antibody against β barrel 1 domain (Ig036, Zedira, Germany). In addition, NF-ĸB p65 was recognized by an antibody from Santa Cruz Biotechnology (Milan, Italy) in the same samples, but it was not detected in MCF-7 because of the low level of expression. Actin was employed as control.

Experiments with NC9 were performed on BrCa cells to verify whether the action of Doxo depends on an accumulation of TG2 as in other types of cancers 45-49. In this approach NC9 was added at 30 μM concentration (K_i_ value) for 16 h, after 24 h of treatment with Doxo for a total exposure time of 40 h. In this way we studied the effects of this drug combination on the expression of NF-ĸB in MCF-7 and MDA-MB-231 cells. Firstly, analysing TG2 protein by Western Blot under conditions allowing discrimination of molecular weight differences, we were able to observe an accumulation of the canonical isoform (78 kDa), but also of an additional band corresponding to the weight of the truncated TGH (62 kDa), with high intensity in cells induced by Doxo, using an antibody reactive against both isoforms (Figure 7). The combined use of Doxo and NC9 decreased both isoforms in each type of cell. Regarding NF-ĸB p65, it decreased effectively in MDA-MB-231 cells, which contain high levels of this factor, in agreement with its role in supporting inflammation in this specific type of tumour, while no conclusion can be reached in MCF-7 in which this factor is undetectable.

**Figure 8:**
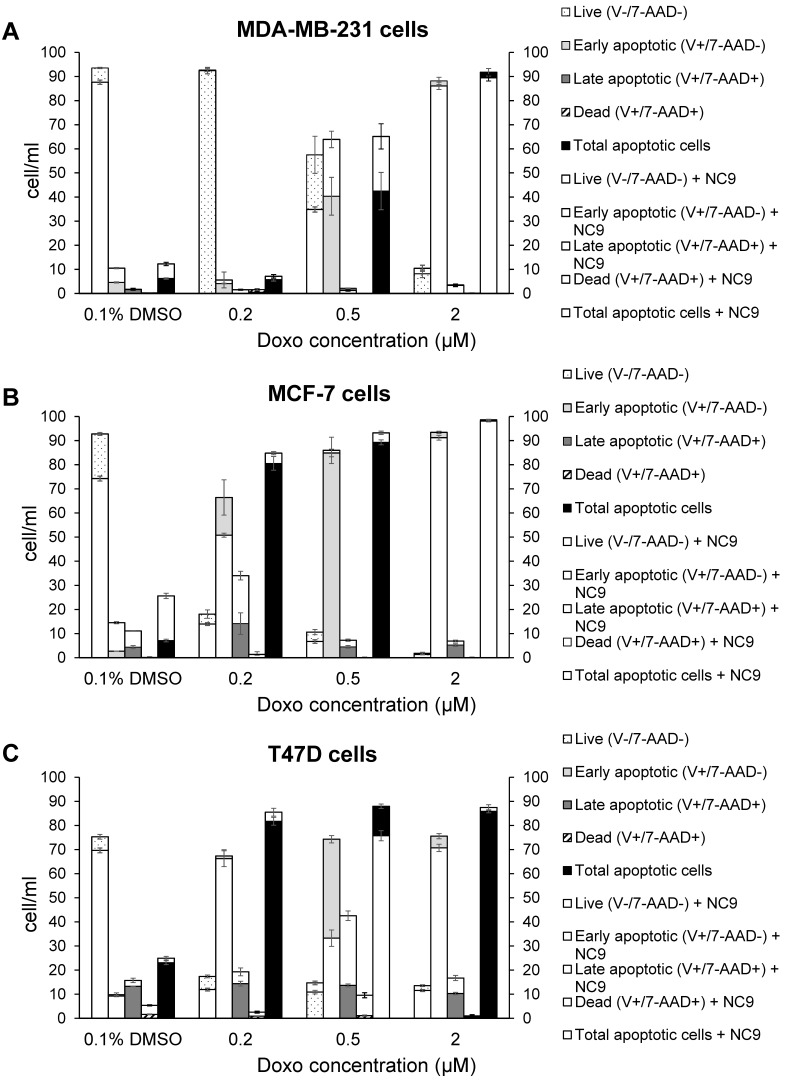
** Effects of NC9 on apoptosis of Doxo treated BrCa cells.** Doxo administration and NC9 treatment were performed under the exact same conditions as in Figure 7. Apoptotic effects were evaluated with Annexin V and MUSE cell analyzer (Luminex Corporation) in MDA-MB-231 (**A**), MCF-7 (**B**) and T47D (**C**) cells. In the charts Live (Annexin V-/7-ADD-), early apoptotic (Annexin V+/7-ADD-), Late apoptotic (Annexin V+/7-ADD+) and dead (Annexin V+/7-ADD+) cells are indicated for each sample. White histograms represent samples treated also with NC9.

Likewise, we performed experiments to analyse apoptosis, since it has been reported that Doxo promotes and TG2 prevents apoptosis in epidermal growth factor activated BrCa cell lines 50, as well as an increase of TG2 has been associated with drug-resistance 52. We analyzed the effects of NC9 on apoptosis induced using several Doxo concentrations, in the presence or absence of the TG2 inhibitor, by assaying after 40 h, of which the last 16 h represent combined treatment. The different phases of apoptosis were detected with Annexin V kit in the triple negative MDA-MB-231 and in ER+/PR+/HER2-MCF-7 and T47D cell lines, both of which can develop drug-resistance. Along the dilution points of Doxo we observed that its double-combination with NC9 drove MDA-MB-231 cells to apoptosis by increasing early apoptosis (+23.55% at 0.5 μM Doxo+30 μM NC9, Figure 8A), while it enhanced late apoptosis in both MCF-7 cells (+19.90% at 0.2 μM Doxo+30 μM NC9, Figure 8B) and T47D cells (+28.8% at 0.5 μM Doxo+30 μM NC9, Figure 8C), under conditions in which the cells were normally highly viable (not less than 10% difference compared to untreated) (Supplementary Figure 3). A decrease of viability was more evident in the T47D cells, but at combined treatment of NC9 at 2 μM Doxo, in which it achieved 37%. Overall, we can conclude that the effects of the combined treatment pushes towards apoptosis some cells that could escape from Doxo therapy. Parallel analysis of cell cycle displayed that double-treatment had different effects depending on the type of cells. In MDA-MB-231 cells, double treatment increased the percentage of cells in G0/G1 with a reduction also of those in S phase, but it almost completely stopped MCF-7 in G2/M. In T47D cells, the addition of NC9 showed an increase of percentage of cells with an accumulation in cycle phase more like MDA-MB-231 cells (Supplementary Figure 4).

## Discussion

Involvement of TG2 in BrCa, postulated since 1996 51, has been confirmed by many reports supporting the relevant role of the enzyme in promoting EMT, metastatic progression 16 and drug resistance 4,52. Since the mechanisms underlying these pathological processes and the relationship of TG2 with transcriptional factors that regulate gene expression and other players supporting these networks have not been sufficiently elucidated, we have tried to highlight interconnections among all these elements. A main point we have considered is the distribution of TG2 isoforms in BrCa cell lines, in relationship with the degree of malignancy. The truncated transcript TGH accumulates in triple negative MDA-MB-231 cells, typically actively transcribing with elevated proliferation rate 2, and higher TG2 protein than MCF-7 cells. This specific accumulation of the truncated isoform TGH likely depends on the stall/interference of RNApol II during transcription, promoting the use of an alternative polyadenylation site along the *TGM2* gene, as already described for other types of cancer cells 14. We have expanded the investigation to a great number of BrCa lines (about 40) confirming that the TGH variant accumulates heavily in the triple negative phenotype, deriving from primary tumours (BT20, HCC1937, Hs578T, SUM149, SUM159, BT549) or metastatic cells isolated from pleural effusions (MDA-MB-231, MDA-MB-436, SUM185, SUM229, SUM1315) that display an aggressive behaviour. In this context, the ratio between full-length TG2 and TGH isoforms could be a relevant feature of actively proliferating cells.

In this perspective we have further analysed the effects of chemotherapeutic agents on the expression of TG2 and of the variant TGH. All the employed drugs upregulated both transcripts in MCF-7 cells, especially AZ5363 53, XL765 54 and Doxo which also induced a significant increase of the coded isoforms, as appreciable in western bot analysis. In the case of MDA-MB-231 cells only Doxo induces *TGM2* transcripts, but we did not observe a further increase in the protein aliquot considering the high levels already present. This drug is largely used in the treatment of solid tumours, but it is known that Doxo induces persistent activation of TG2 in association with cell survival and resistance to therapy 25.

Considering the mechanism of Doxo, it binds selective DNA sequences affecting methylation at CpG islands also at selected genes associated to cell survival 37 and interfering with activity of Topoisomerase II, producing truncated variants 22-24. Since our results are in line with interference of Doxo with the *TGM2* gene, we scrutinized involvement of transcriptional factors with regulatory functions, considering that Doxo resistant cells do not display *TGM2* silencing associated with demethylation events 34. We focussed on some specific promoter regions, including targets of methylation/acetylation and sequences recognized by factors associated with Doxo-resistance 35 and at a portion of intron 10 located after the stop codon leading to truncated TGH 14 in experiments of *in vitro* arrested-PCR carried out in the presence of the drug. The results demonstrated that Doxo inhibited the amplification of mimic templates.

Of special interest is the presence of GATA3 sites both in the transcription starting region and in the intron 10 close to sites for RNApol II, which suggests a possible co-action of GATA3 and Doxo in the regulation of this gene 43. To clarify this point, we silenced GATA3 in MCF-7 cells (which already express this factor) and in MDA-MB-231 upon stimulation with Doxo, because these cells express very low levels unless treated. Under these conditions, siRNA against GATA3 downregulates the full-length TG2 in MCF-7 cells and TGH in MDA-MB-231 cells (which are hypomethylated at the *TGM2* promoter and present high levels of TGH). All these data confirm a relationship among Doxo, GATA3 and the *TGM2* expression, by one side influencing GATA3 (which becomes downregulated in MCF-7 and upregulated in MDA-MB-231 cells) by the other modulating the levels of transglutaminase isoforms, which likely act in transducing downstream cascade(s).

**Figure 9:**
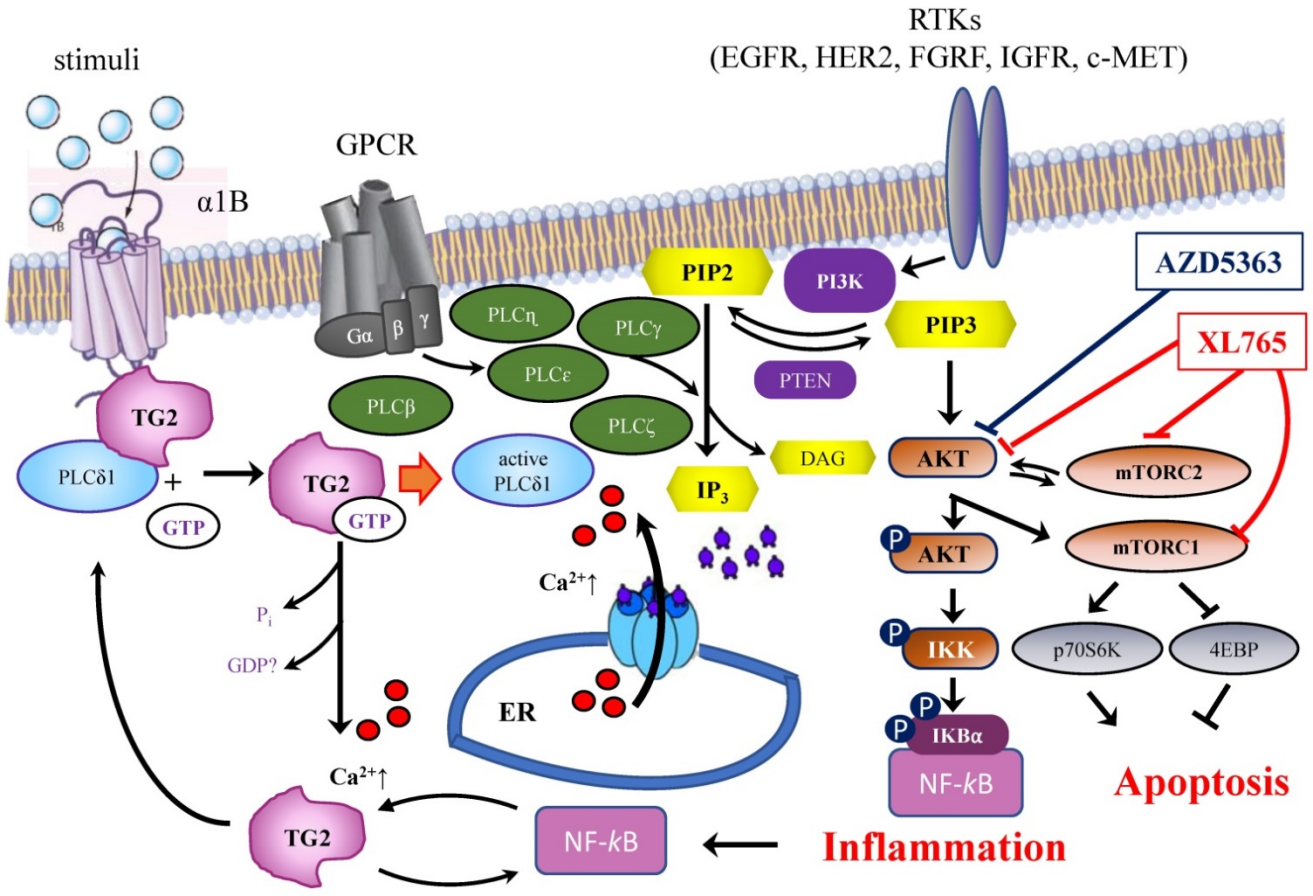
Schematic representation of the interrelations among TG2, PLCδ1 and PIP2/PIP3 signaling pathways and target proteins of ADZ5363 and XL765 anticancer agents.

A correlation between *TGM2* gene and tumour behaviour has been proposed in several studies on ovarian 17 and pancreatic tumours 19 in a variety of experimental systems 21. The results suggested that the downregulation of TG2 can reverse EMT and modulate the sensitivity of BrCa toward anticancer drugs. These line of experimental evidences were supported using inhibitors mainly directed against the transamidating activity of TG2, but in some instances also affecting the G-protein signalling 50. This is the case of the well-known irreversible inhibitor NC9, which has been shown to prevent nuclear translocation of TG2 and increase its proteasome-mediated degradation 55, altering conformation of TG2 enzyme thus blocking both catalytic activity and intracellular GTP-binding 47,56. The latter feature of NC9 is particularly relevant, if we consider that the TGH isoform of the enzyme is still capable of hydrolysing GTP, albeit with low binding efficiency 12. NC9 has been shown to irreversibly block both functions, especially in cancer types 45,47-49,55,56. Our data demonstrated that the choice to use NC9 in combined treatments proved effective, driving MDA-MB-231 cells to early apoptosis and MCF-7 and T47D cells to late apoptosis, limiting the progression of cell cycle but inducing accumulation of cells in different phases, depending on the type of cell, while also decreasing NF-ĸB in MDA-MB-231 cells. In agreement with data from other types of cancer, NC9 attenuated NF-ĸB-mediated inflammation promoted by TG2 upregulation under stimulus by ATRA, opposing its nuclear translocation 55. Analogously, it has been reported that in MDA-MB-231 cells and in MCF-7 cell resistant to Doxo, the activation of NF-ĸB correlates with TG2 inhibition by siRNA, increasing IĸBα with accumulation of NF-ĸB in the cytosol 18. Notably, BrCa is known to be supported by the inflammatory processes 18 and NF-ĸB itself promotes overexpression of *TGM2* by a positive feedback loop and by TG2-mediated production of interleukins 8,57. Hence TG2 inhibitors can provide an advantage to control the enzymatic activity, when this condition occurs, as in the case of BrCa treatment with chemotherapeutic agents, like AZD5363 or XL765, interfering with pathways involving TG2 (Figure 9). In our studies, the responses to AZD5363 and to XL765 were different in MDA-MB-231 and MCF-7 cells, likely related to the different mechanisms of action of these compounds 53,58. The relationship between aggressive phenotype in triple negative BrCa cells and the PLCδ1 pathway has been also associated with poor prognosis, highlighting its value as therapeutic target 58. Our observations in several triple negative cell lines suggest a correlation between these features and an unbalanced ratio among TG2 isoforms, whose functional activity must be better defined.

TG2 is an important predictive and prognostic factor 4,59 and analysis of the gene expression profile of *TGM2* can be useful to test potential effects for evaluation of efficacy/resistance to therapy in patients with BrCa, performing predictive *in vitro* experiments before setting up *in vivo* administration. Here, we propose that the monitoring of *TGM2* variants and the use of appropriate strategies to suppress *TGM2* expression (with antisense-RNA or RNA interference), and the use of enzyme inhibitors, can improve through a combined action the effects of common anticancer agents. In this perspective, inhibition TG2 could represent a therapeutic strategy to drive the cells to apoptosis and to oppose drug-resistance 50,60. This might open the way to a fruitful field of investigation for novel therapeutic combinations, in which TG2 inhibition could be associated with integrin 61 and mTOR inhibitors 62 or strategies targeting transcription factors, for instance directed to GATA3, effective against mRNA 63 or the DNA-binding activity of this factor 64.

## Supplementary Material

Supplementary figures and table.

## Abbreviations

BrCa: breast cancer
CT: threshold cycle
Doxo: Doxorubicin
EMT: epithelial-mesenchymal transition
FFPE: formalin-fixed paraffin-embedded
FBS: foetal bovine serum
HER2: Epidermal Growth Factor Receptor 2
HPRT: hypoxanthine phosphoribosyltransferase 1
LEV: last exons variant
lncRNA: long non-coding RNA
NI: normalized signal intensity
RT-qPCR: reverse transcription and quantitative polymerase chain reaction
siRNA negative control): siRNA neg
TG2: Transglutaminase type 2
*TGM2*: Transglutaminase 2 gene
