# Supplementary material for: Dysregulation of Transglutaminase type 2 through GATA3 defines aggressiveness and Doxorubicin sensitivity in breast cancer
Source: Int J Biol Sci. 2022 Jan 1;18(1):1–14. doi: 10.7150/ijbs.64167 (PMC8692156; doi:10.7150/ijbs.64167)
Supplement: Supplementary file 1 — Supplementary figures and table. [file ijbsv18p0001s1.pdf]

Table S1

	Sequence of DNA fragment	PCR size (bp)
PCR1	aaagcctcaaggtggagacaaaggtgaatatttagaaactttggaataacccactaccagc<br>cagatattccatttaaacctcacagtatgattcttatcatttgttaaatgaggaaccctgggtt<br>cagagaggggaagtgcctcacccaaggccacacagtcaggaagtgggaaagcagggcct<br>gtgctggctccgccaaggttcagaggtcagtcctgtctccccgtgccctgcagggcagtcctc<br>aggggcctgggccatgtccagttctacagcttttagcaagtgaggcctccgaggactgcagc<br>ttgtgctcctaacacatggcatg	332
PCR2	tctcactgtgtagccatgtccactacacagcaagtcacttaacctctgtgcctcagtttcctca<br>tttgtaaaatgggggcagtattggtacccactccccagagtgtcatgaggactgagtgagct<br>aattcatggagagtttgtacagcagcagccaaacagtcagccctcaatggatgctggctgt<br>gagaagaatcccattagattattcctggatcccagcacagggcttagcacagaattccagg<br>cctgtcccgcacccagccagctgcccccacttcctctcccacattccacagaactatggcca<br>cctgagtgacgcatggcgttgagcaaccccaggatgtggccagccggggtcaggactggct<br>tctaggaagttggcagacagaggccagaacccaggcttggcccagagacctggattccagt<br>cccagtgaccaggcacccttgcccctctctgggtttcagttttcccatttgttcaaagggactg<br>ggaaactgtgccctgtcttacctcaccggggctgtaaaggtttcaaaactcccctgagtccc<br>agagagggtgatcatggcct	578
PCR3	ctccacatctgtgtgtccaggtgcaccccggttccgttgtgtgtttctatgagggtgctgcgtg<br>tgtctgtatctgagtgtgtgtgtccaggtgtctgttctccaaggtctgagtctgtgggtccagg<br>tgtgtctgtatcctgggctagttgtgtgtccctgtc**cgctcccccagggggcgccctcgtccgg**<br>**ccgccgtccctccctcgggctccggtcccctgggtgagccccagcgctggcggcgtgggccc**<br>**gggactgggcaatgggtggcctcccaggtcgccgccttcccgcggggccccgcccccggcc**<br>**cgccccaaagcgggctataagttagcgccgctctccgcctcggcagtgccagccgccagtg**<br>**gtcgcacttggagggtctcgccgccagtggaaggagccaccgcccccgcccgaccatggc**<br>**cgagggtgagtatcagagtcaccgcagccgccactcaggggcccccg**gggcattgggggtc<br>aggacccggtgggagctgaatccctaagtcctgatttggagagcaacaggcag	549
PCR4	aagctgagatgcgttccctctaagcccccatactgtggttgagttacttgttattctttgatga<br>taagatctggggaatttccgtgtgctggtggacaggaagccaggccctggatggctgaaag<br>gaatccagttgccccc	141

Fig. S1

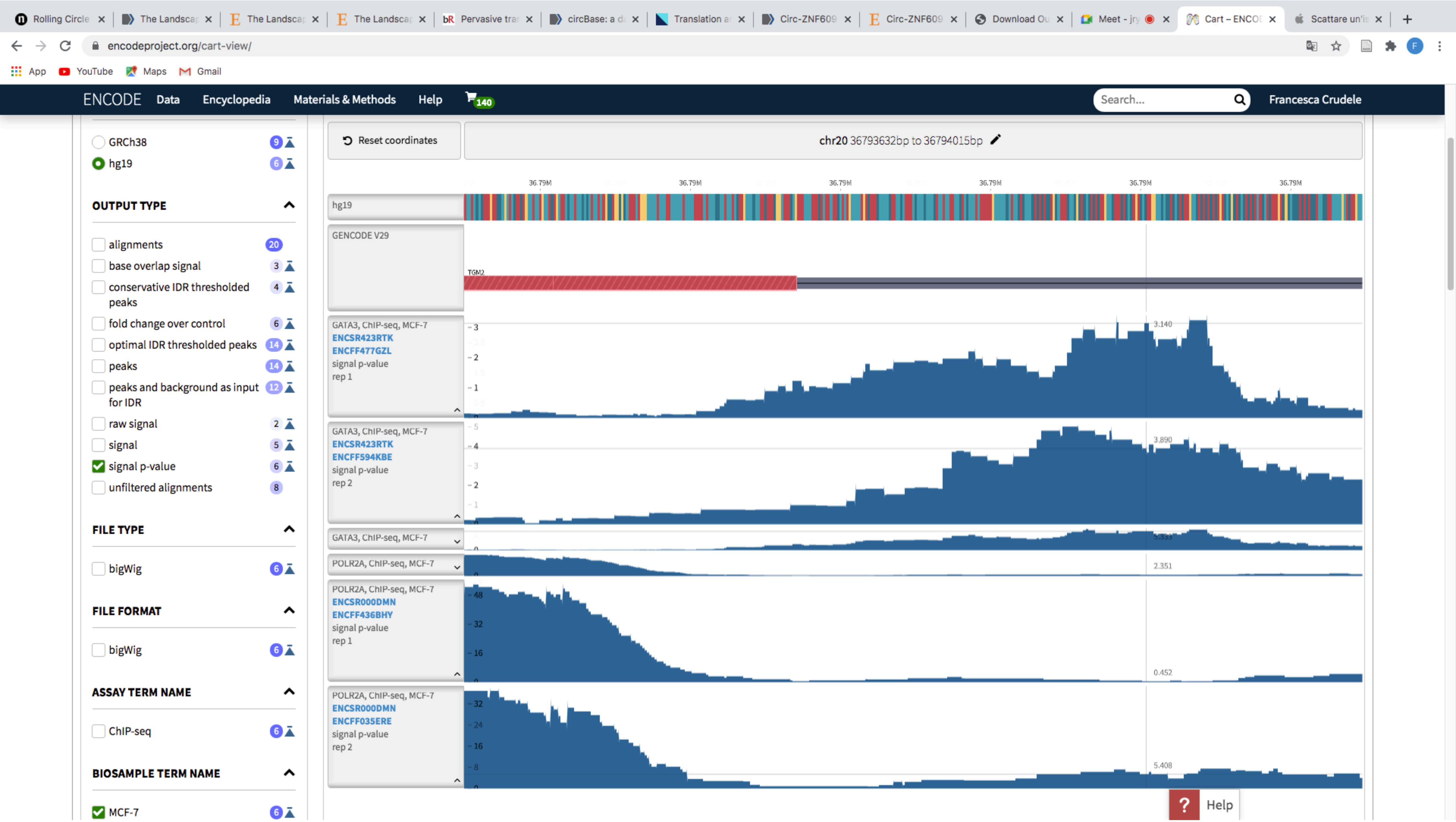

encodeproject.org/cart-view/
ENCODE
Data
Encyclopedia
Materials & Methods
Help
Francesca Crudele
GRCh38
hg19
OUTPUT TYPE
alignments
base overlap signal
conservative IDR thresholded peaks
fold change over control
optimal IDR thresholded peaks
peaks
peaks and background as input for IDR
raw signal
signal
signal p-value
unfiltered alignments
FILE TYPE
bigWig
FILE FORMAT
bigWig
ASSAY TERM NAME
ChIP-seq
BIOSAMPLE TERM NAME
MCF-7
Reset coordinates
chr20 36793632bp to 36794015bp
hg19
GENCODE V29
TGM2
GATA3, ChIP-seq, MCF-7
ENCSR423RTK
ENCFF477GZL
signal p-value
rep 1
GATA3, ChIP-seq, MCF-7
ENCSR423RTK
ENCFF594KBE
signal p-value
rep 2
GATA3, ChIP-seq, MCF-7
POLR2A, ChIP-seq, MCF-7
POLR2A, ChIP-seq, MCF-7
ENCSR000DMN
ENCFF436BHY
signal p-value
rep 1
POLR2A, ChIP-seq, MCF-7
ENCSR000DMN
ENCFF035ERE
signal p-value
rep 2
Help

Fig. S1

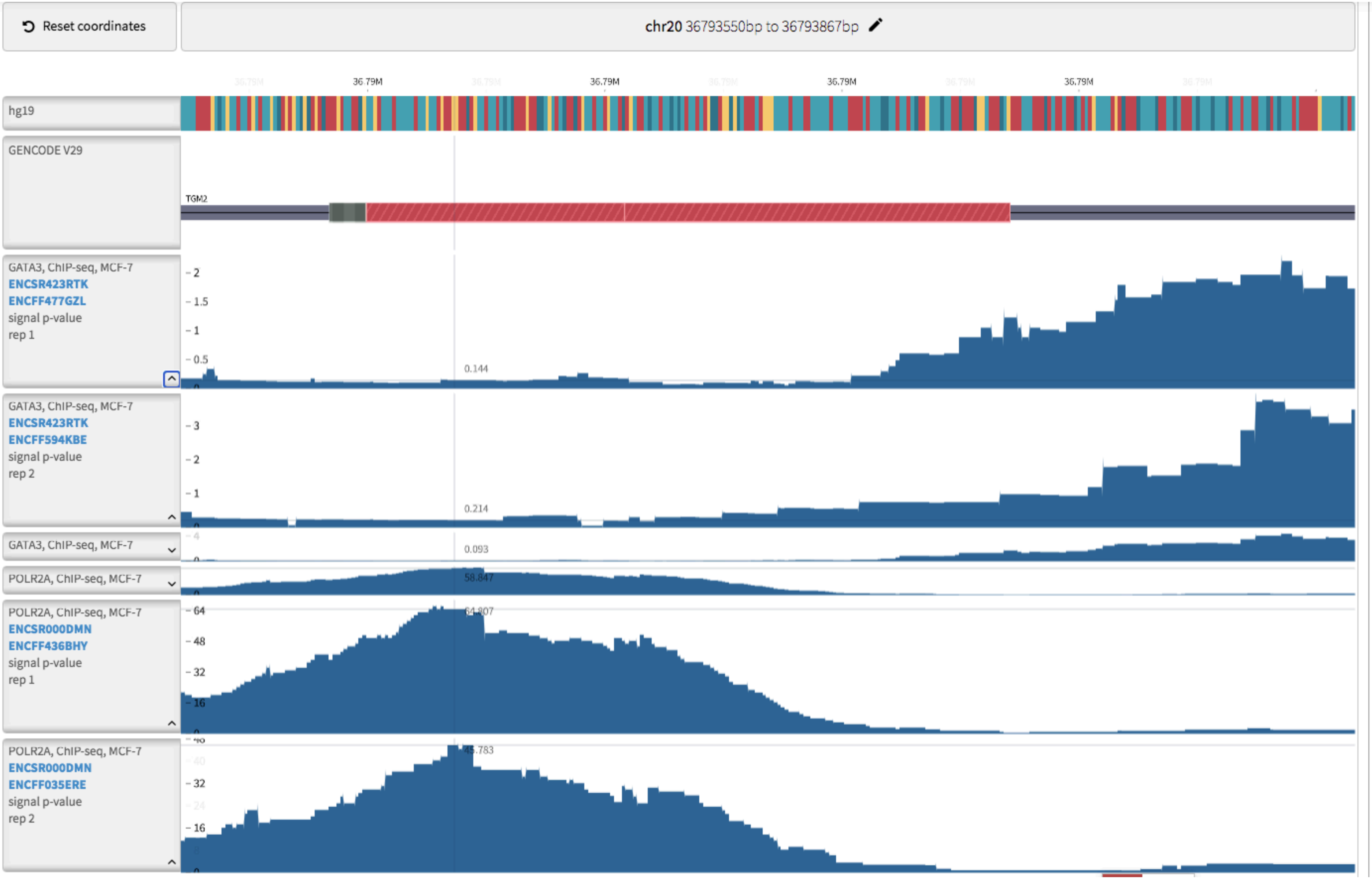

Reset coordinates
chr20 36793550bp to 36793867bp
36.79M
36.79M
36.79M
36.79M
hg19
GENCODE V29
TGM2
GATA3, ChIP-seq, MCF-7
ENCSR423RTK
ENCFF477GZL
signal p-value
rep 1
2
1.5
1
0.5
0.144
GATA3, ChIP-seq, MCF-7
ENCSR423RTK
ENCFF594KBE
signal p-value
rep 2
3
2
1
0.214
GATA3, ChIP-seq, MCF-7
0.093
POLR2A, ChIP-seq, MCF-7
58.847
POLR2A, ChIP-seq, MCF-7
ENCSR000DMN
ENCFF436BHY
signal p-value
rep 1
64
48
32
16
64.807
POLR2A, ChIP-seq, MCF-7
ENCSR000DMN
ENCFF035ERE
signal p-value
rep 2
32
16
45.783

Fig. S2

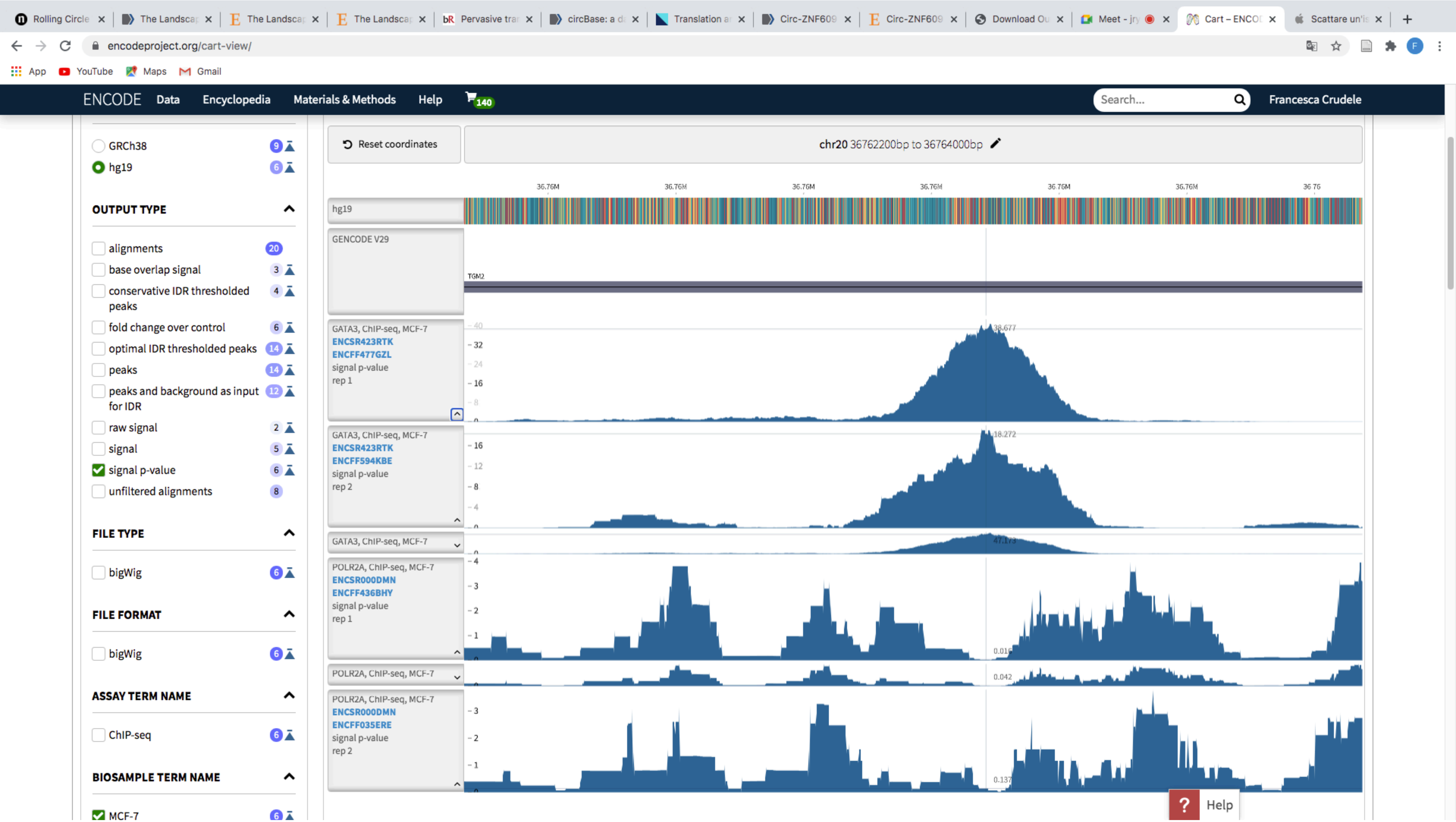
encodeproject.org/cart-view/
ENCODE
Data
Encyclopedia
Materials & Methods
Help
140
Search...
Francesca Crudele
GRCh38 9
hg19 6
OUTPUT TYPE
alignments 20
base overlap signal 3
conservative IDR thresholded peaks 4
fold change over control 6
optimal IDR thresholded peaks 14
peaks 14
peaks and background as input for IDR 12
raw signal 2
signal 5
signal p-value 6
unfiltered alignments 8
FILE TYPE
bigWig 6
FILE FORMAT
bigWig 6
ASSAY TERM NAME
ChIP-seq 6
BIOSAMPLE TERM NAME
MCF-7 6
Reset coordinates
chr20 36762200bp to 36764000bp
hg19
GENCODE V29
TGM2
GATA3, ChIP-seq, MCF-7
ENCSR423RTK
ENCFF477GZL
signal p-value
rep 1
GATA3, ChIP-seq, MCF-7
ENCSR423RTK
ENCFF594KBE
signal p-value
rep 2
GATA3, ChIP-seq, MCF-7
POLR2A, ChIP-seq, MCF-7
ENCSR000DMN
ENCFF436BHY
signal p-value
rep 1
POLR2A, ChIP-seq, MCF-7
POLR2A, ChIP-seq, MCF-7
ENCSR000DMN
ENCFF035ERE
signal p-value
rep 2
Help

Fig. S3

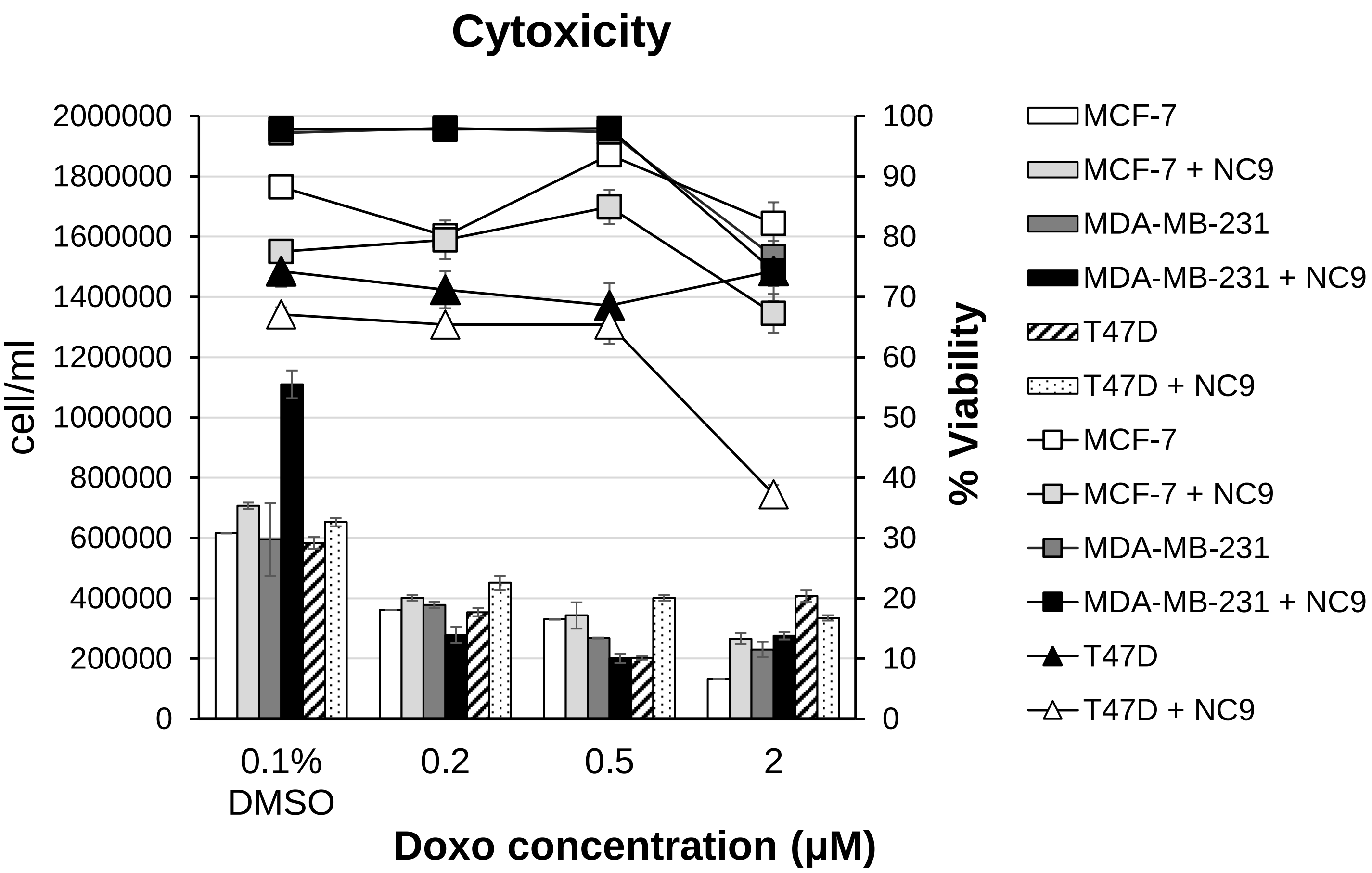
Cytoxicity
2000000
1800000
1600000
1400000
1200000
1000000
800000
600000
400000
200000
0
cell/ml
100
90
80
70
60
50
40
30
20
10
0
% Viability
0.1% DMSO
0.2
0.5
2
Doxo concentration (µM)
MCF-7
MCF-7 + NC9
MDA-MB-231
MDA-MB-231 + NC9
T47D
T47D + NC9
MCF-7
MCF-7 + NC9
MDA-MB-231
MDA-MB-231 + NC9
T47D
T47D + NC9

Fig. S4

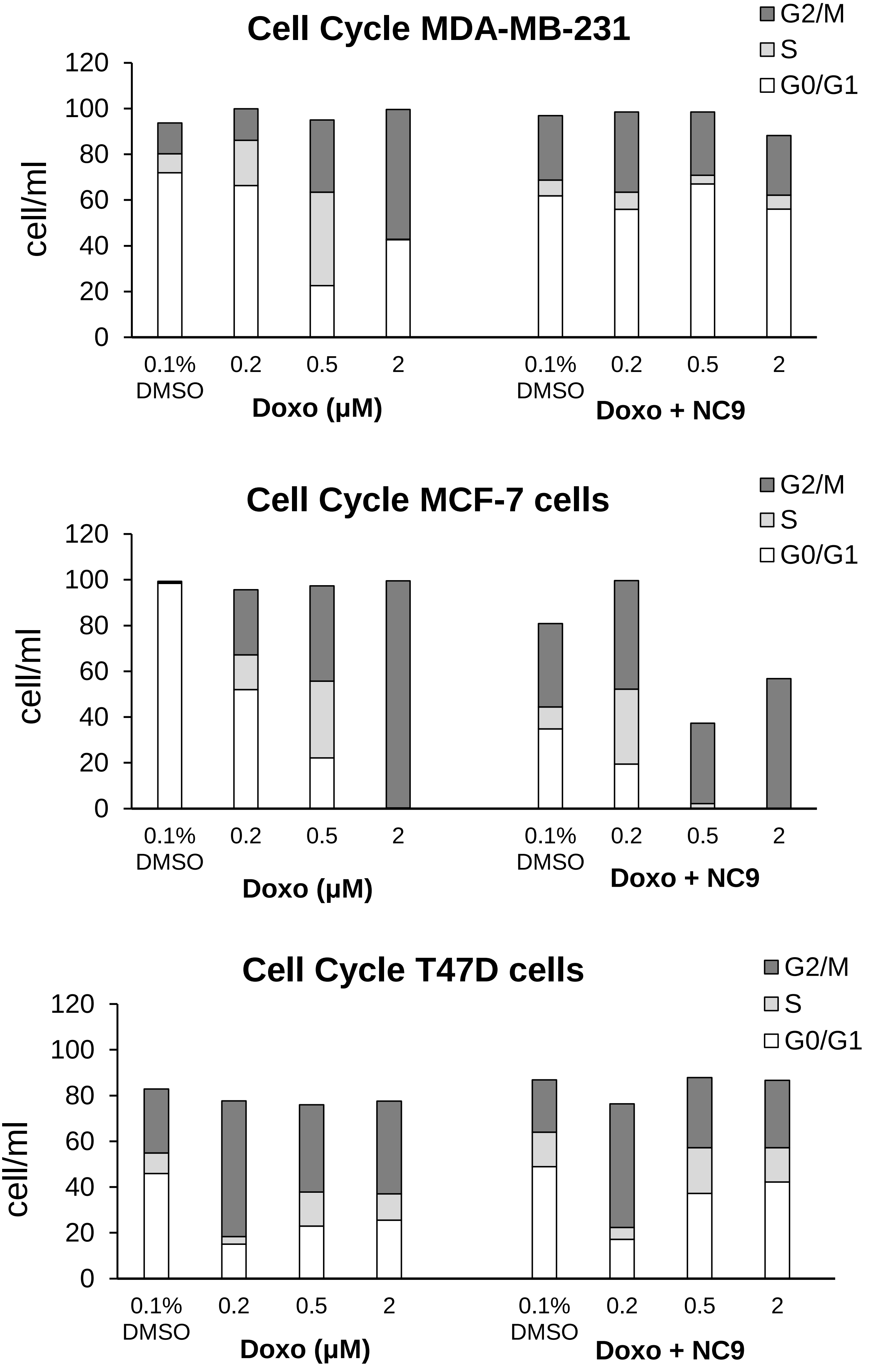
Cell Cycle MDA-MB-231
G2/M
S
G0/G1
cell/ml
0
20
40
60
80
100
120
0.1% DMSO
0.2
0.5
2
Doxo (μM)
0.1% DMSO
0.2
0.5
2
Doxo + NC9
Cell Cycle MCF-7 cells
G2/M
S
G0/G1
cell/ml
0
20
40
60
80
100
120
0.1% DMSO
0.2
0.5
2
Doxo (μM)
0.1% DMSO
0.2
0.5
2
Doxo + NC9
Cell Cycle T47D cells
G2/M
S
G0/G1
cell/ml
0
20
40
60
80
100
120
0.1% DMSO
0.2
0.5
2
Doxo (μM)
0.1% DMSO
0.2
0.5
2
Doxo + NC9
